# Supplementary material for: Type I Cystatin Derived from Fasciola gigantica Suppresses Macrophage-Mediated Inflammatory Responses
Source: Pathogens. 2023 Mar 1;12(3):395. doi: 10.3390/pathogens12030395 (PMC10051455; doi:10.3390/pathogens12030395)
Supplement: Supplementary file 1 [file pathogens-12-00395-s001.zip › pathogens-2144793-supplementary.pdf]

Supplementary materials

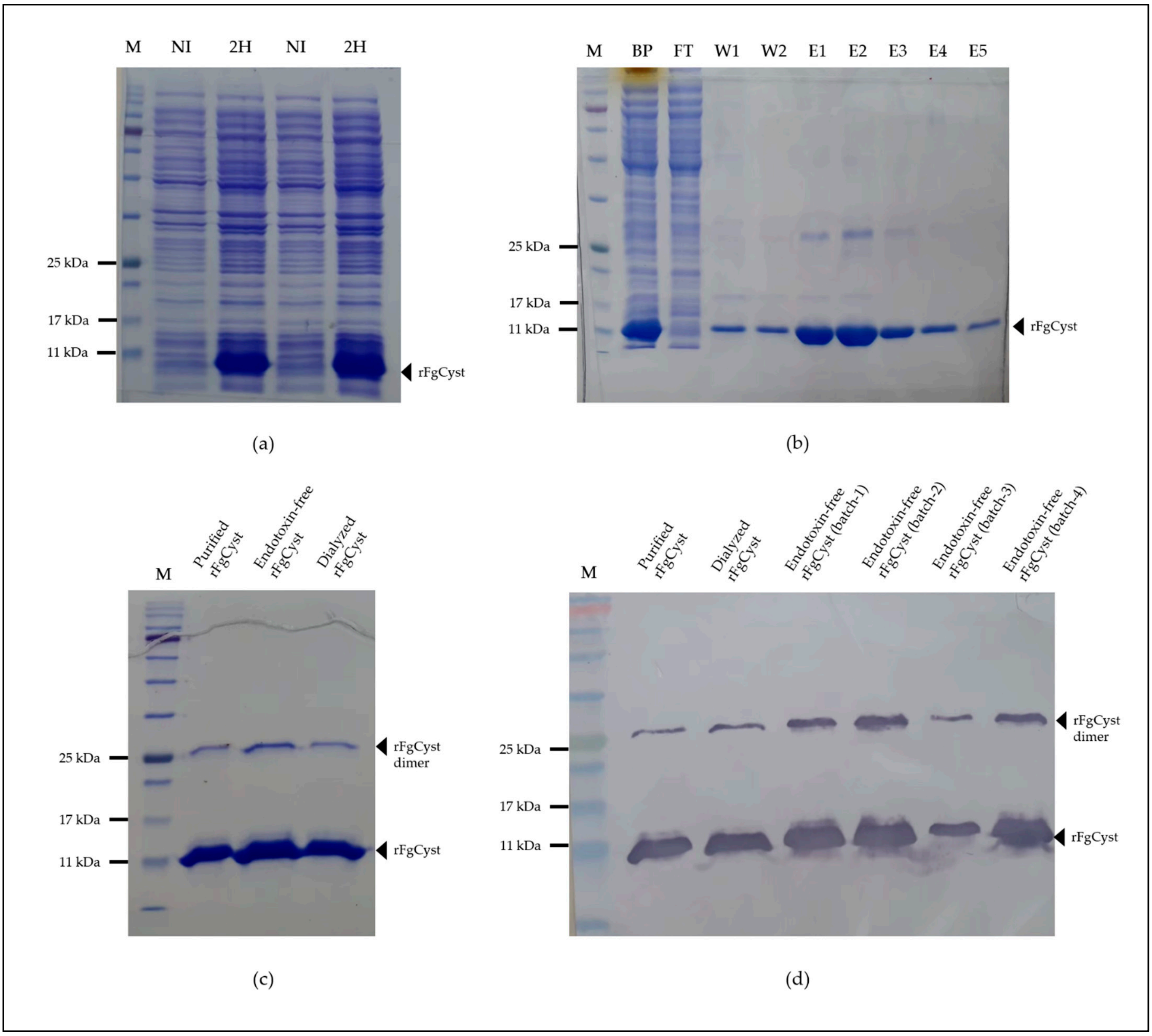

Figure S1. Production of rFgCyst (**a**) SDS-PAGE of M15 *E. coli* containing pQE-30/FgCyst (two clones) demonstrated non-induction (NI) and 2 h induction with IPTG (2H); (**b**) SDS-PAGE of purified rFgCyst under native condition; (**c**) SDS-PAGE of purified, endotoxin-free, and dialyzed rFgCyst; (**d**) Western analysis of purified, endotoxin-free, and dialyzed rFgCyst. M: Tricolor Broad Range Prestained Protein Ladder (Vivantis, Malaysia); BP: before purifying; FT: flow through; W1: wash fraction-1; W2: wash fraction-2; E1-E5: elution fraction 1-5.

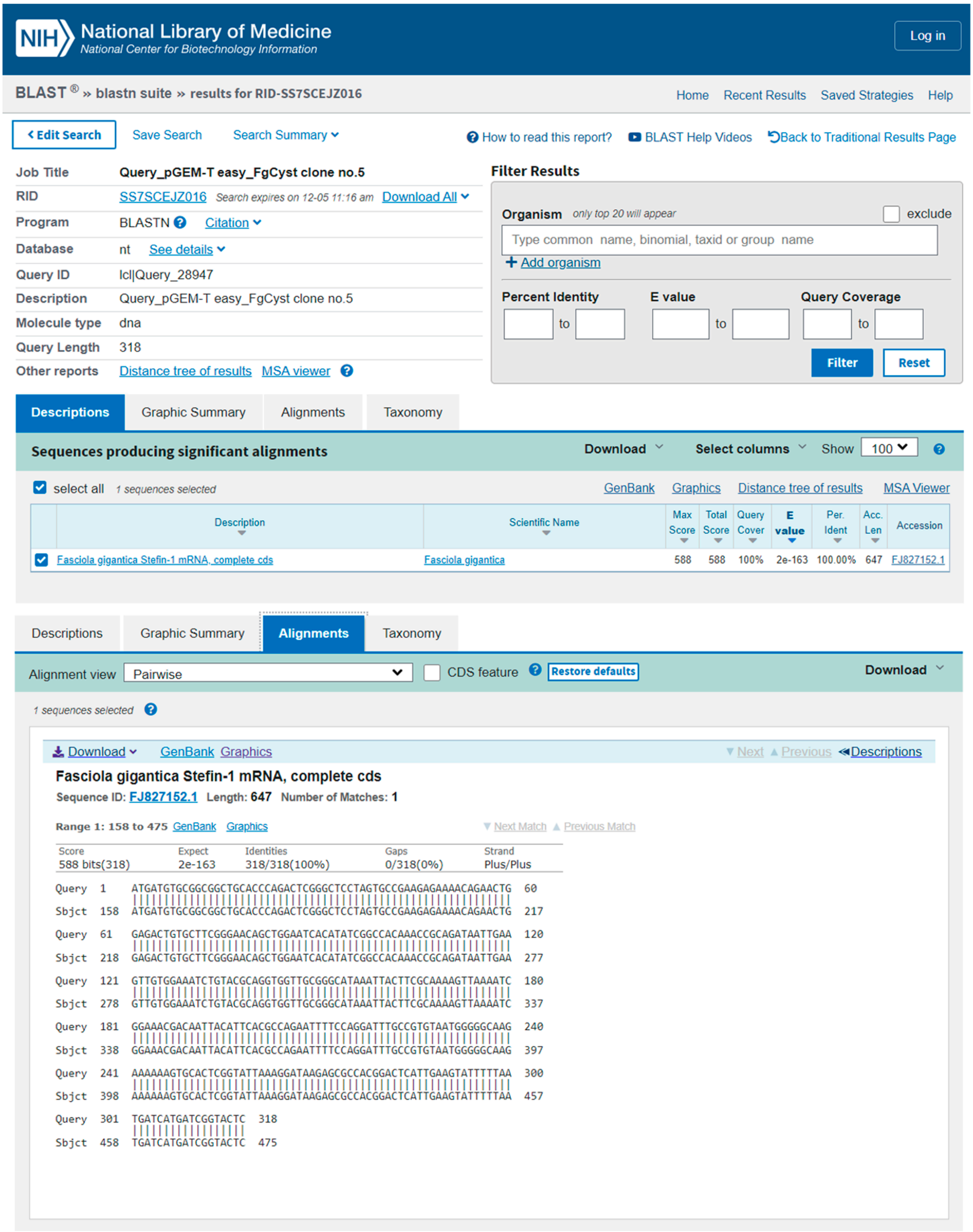

Figure S2. Sequence alignment of the cloned-FgCyst from this study to the database showing 100% identity without signal peptide and 3′ untranslated region.
